# Supplementary material for: Accelerated Endothelialization of Nanofibrous Scaffolds for Biomimetic Cardiovascular Implants
Source: Materials (Basel). 2022 Mar 9;15(6):2014. doi: 10.3390/ma15062014 (PMC8955317; doi:10.3390/ma15062014)
Supplement: Supplementary file 1 [file materials-15-02014-s001.zip › materials-1605900-supplementary.pdf]

materials

MDPI

Supplementary Materials to Article

Accelerated Endothelialization of Nanofibrous Scaffolds for Biomimetic Cardiovascular Implants

Claudia Matschegewski [1], Stefanie Kohse [2], Jana Markhoff [2], Michael Teske [2], Katharina Wulf [2], Niels Grabow [2], Klaus-Peter Schmitz [1,2] and Sabine Illner [2,*]

[1] Institute for ImplantTechnology and Biomaterials e.V., Friedrich-Barnewitz-Straße 4, 18119 Rostock, Germany; claudia.matschegewski@uni-rostock.de, schmitz@iib-ev.de

[2] Institute for Biomedical Engineering, Rostock University Medical Center, Friedrich-Barnewitz-Straße 4, 18119 Rostock, Germany; jana.markhoff@uni-rostock.de; stefaniekohse@web.de; michael.teske@uni-rostock.de; katharina.wulf@uni-rostock.de; niels.grabow@uni-rostock.de

* Correspondence: sabine.illner@uni-rostock.de; Tel.: +49-381-54345-521

Section 1. Introduction

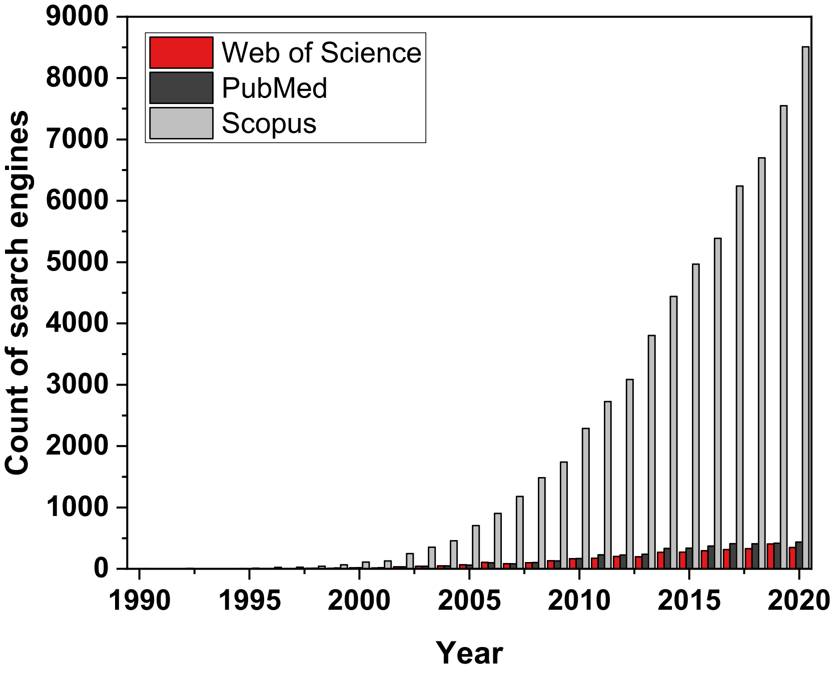

Figure S1: The annual number of publications for the last 20 years with topic "engineer* cardi* scaffold*" provided by the search engine of Scopus, PubMed and Web of Science before the 15th February, 2022.

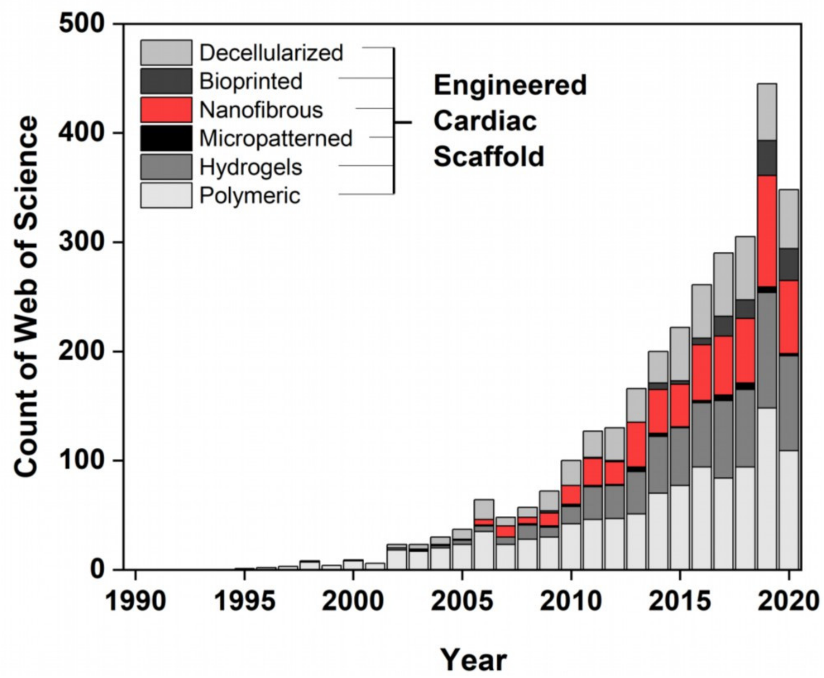

Figure S2: The annual number of publications for the last 20 years with topic "engineer* cardi* scaffold*" associated with supplementary search terms: "polymer*", "hydrogel*", "micropattern*", "nanofib*", "bioprint*" or "decellular*", provided by the search engine of Web of Science before 15th February 2022.

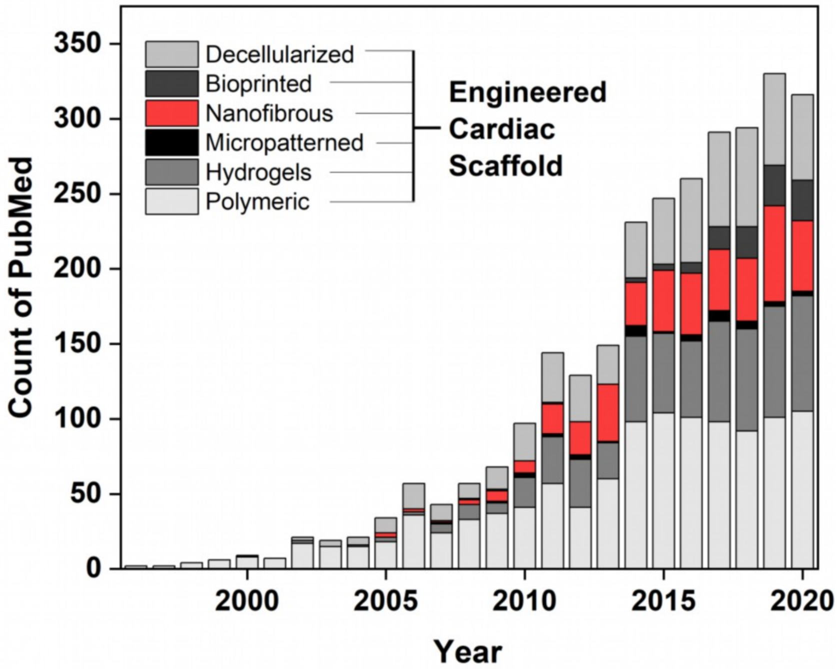

Figure S3: The annual number of publications for the last 20 years with topic "engineer* cardi* scaffold*" associated with supplementary search terms: "polymer*", "hydrogel*", "micropattern*", "nanofib*", "bioprint*" or "decellular*", provided by the search engine of PubMed before before 15th February 2022.

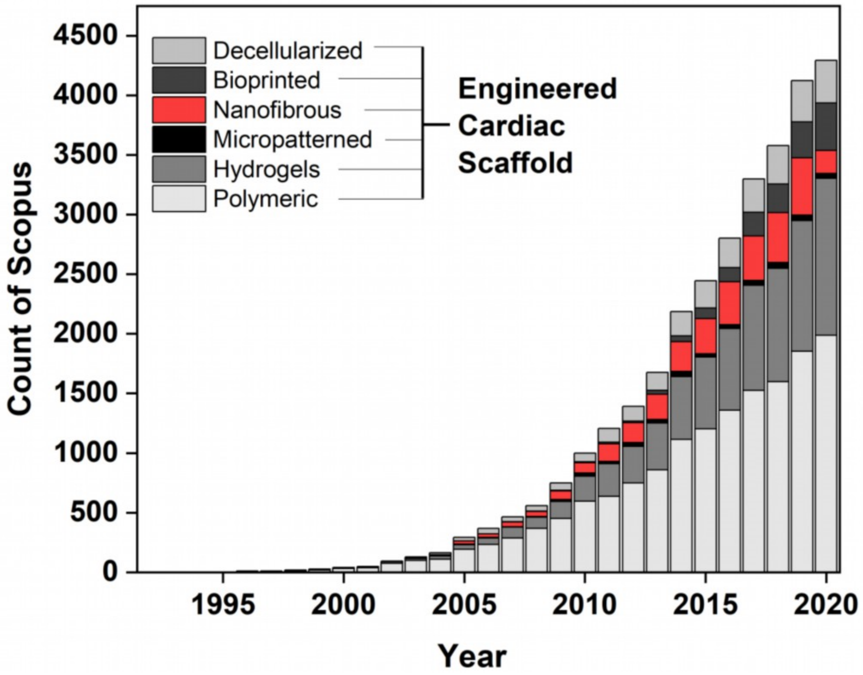

Figure S4: The annual number of publications for the last 20 years with topic "engineer* cardi* scaffold*" associated with supplementary search terms: "polymer*", "hydrogel*", "micropattern*", "nanofib*", "bioprint*" or "decellular*", provided by the search engine of Scopus before 15th February 2022.

Section 3.1. Morphology of polymeric nonwovens

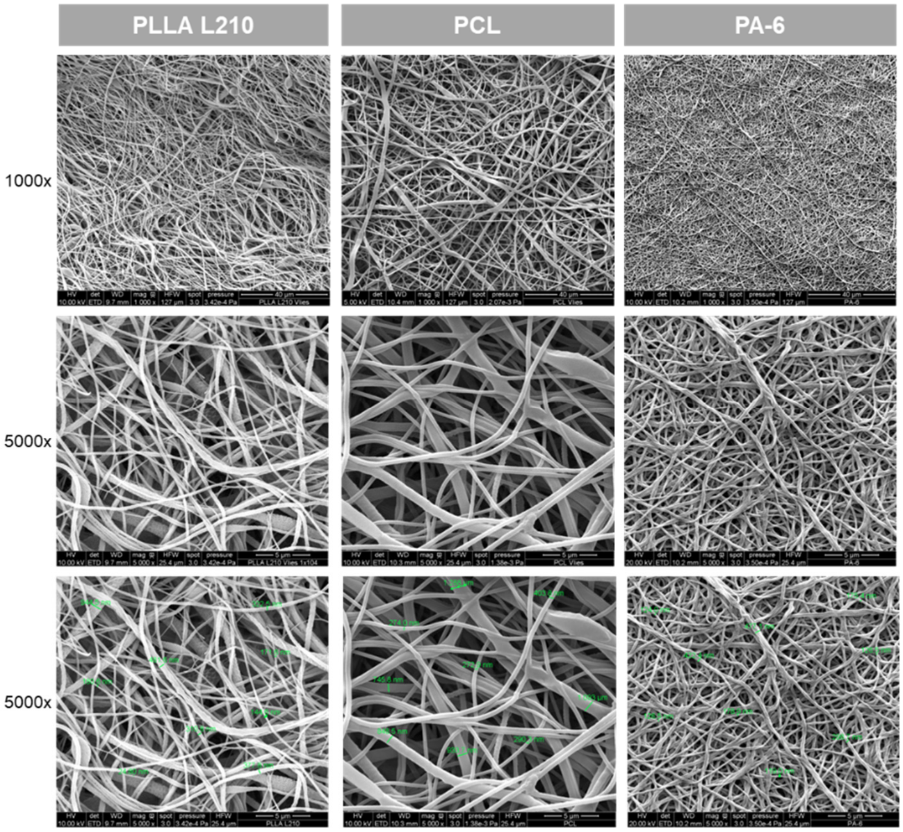

Figure S5: Representative SEM images of PLLA L210, PCL and PA-6 nonwovens at magnification 1000x and 5000x.

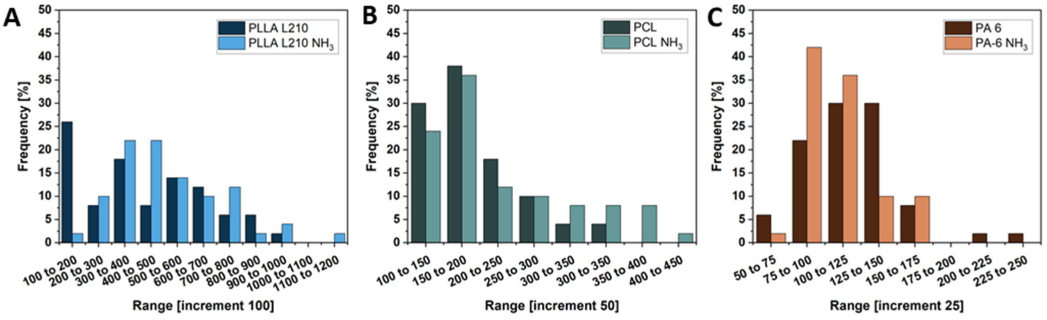

Figure S6: Frequency of the respective fiber diameters within the individually defined increments for untreated and activated (A) PLLA L210, (B) PCL and (C) PA-6 nonwovens based on 50 individual measurement points (where for each polymer 10 fibers were measured manually in 5 SEM images).

Section 3.6. Endothelial actin cytoskeleton formation on polymeric nonwovens

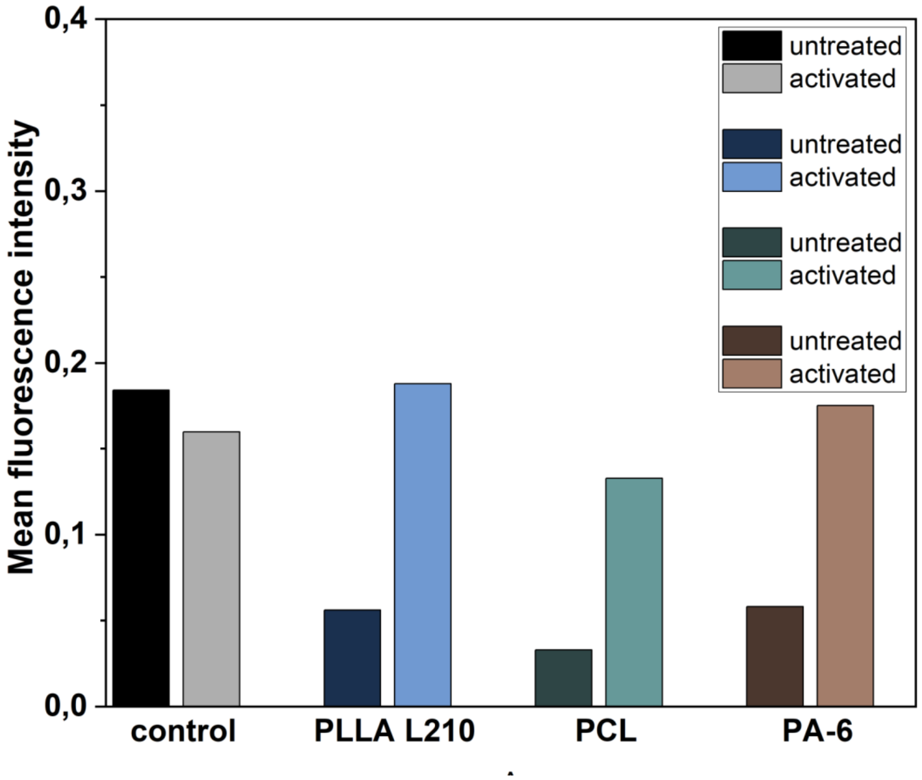

Figure S7. Fluorescence intensity of F-actin formation (phalloidin-TRITC for F-actin) in human endothelial EA.hy926 cells grown for 48 h on untreated and NH_3-plasma functionalized polymeric nonwovens and polystyrene control surface (n = 1).

Section 3.7. Expression of endothelial cell-specific PECAM-1 marker

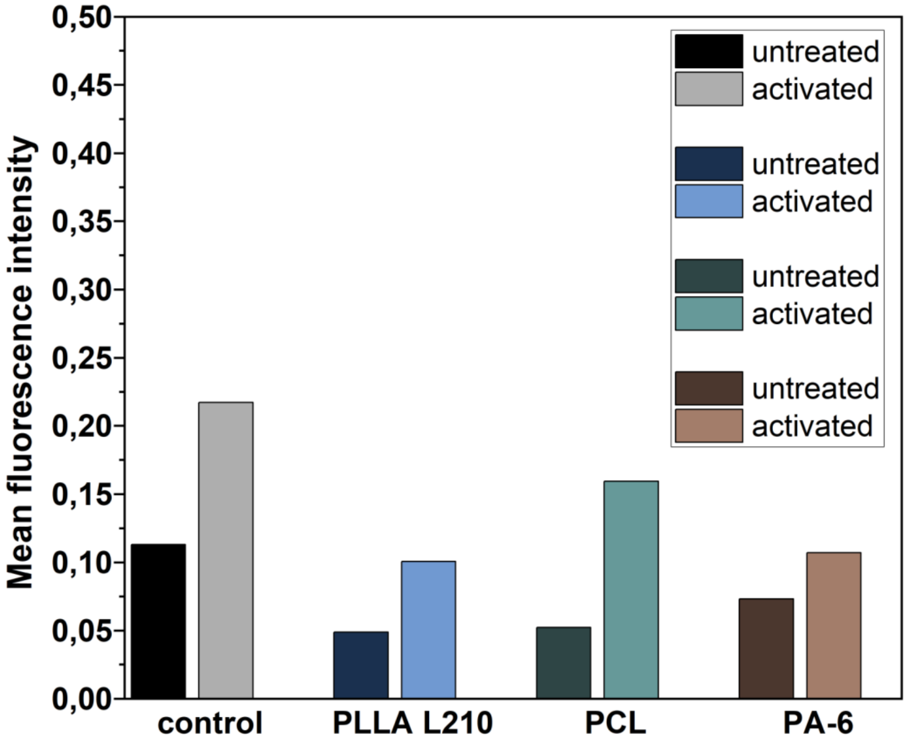

Figure S8. Mean fluorescence intensity of CD 31 expression by human endothelial EA.hy926 cellsgrown for 48 h on untreated and NH_3-plasma functionalized polymeric nonwovens (n = 1).
